# Targeting TLR Signaling Cascades in Systemic Lupus Erythematosus and Rheumatoid Arthritis: An Update

**DOI:** 10.3390/biomedicines12010138

**Published:** 2024-01-09

**Authors:** George D. Kalliolias, Efthimia K. Basdra, Athanasios G. Papavassiliou

**Affiliations:** 1 Hospital for Special Surgery, Arthritis & Tissue Degeneration, New York, NY 10021, USA; kallioliasg@hss.edu; 2 Department of Medicine, Weill Cornell Medical College, New York, NY 10065, USA; 3 Regeneron Pharmaceuticals, Inc., Tarrytown, NY 10591, USA; 4 Department of Biological Chemistry, Medical School, National and Kapodistrian University of Athens, 11527 Athens, Greece; ebasdra@med.uoa.gr

**Keywords:** systemic lupus erythematosus, rheumatoid arthritis, Toll-like receptors, endosomal TLRs, TLR4, TLR antagonists, small molecules, kinase inhibitors, proteolysis-targeting chimeras, peptidomimetics

## Abstract

Evidence from animal models and human genetics implicates Toll-like Receptors (TLRs) in the pathogenesis of Systemic Lupus Erythematosus (SLE) and Rheumatoid Arthritis (RA). Endosomal TLRs sensing nucleic acids were proposed to induce lupus-promoting signaling in dendritic cells, B cells, monocytes, and macrophages. Ligation of TLR4 in synovial macrophages and fibroblast-like synoviocytes (FLSs) by endogenous ligands was suggested to induce local production of mediators that amplify RA synovitis. Inhibition of TLRs using antagonists or monoclonal antibodies (mAbs) that selectively prevent extracellular or endosomal TLR ligation has emerged as an attractive treatment strategy for SLE and RA. Despite the consistent success of selective inhibition of TLR ligation in animal models, DV-1179 (dual TLR7/9 antagonist) failed to achieve pharmacodynamic effectiveness in SLE, and NI-0101 (mAb against TLR4) failed to improve arthritis in RA. Synergistic cooperation between TLRs and functional redundancy in human diseases may require pharmacologic targeting of intracellular molecules that integrate signaling downstream of multiple TLRs. Small molecules inhibiting shared kinases involved in TLR signaling and peptidomimetics disrupting the assembly of common signalosomes (“Myddosome”) are under development. Targeted degraders (proteolysis-targeting chimeras (PROTACs)) of intracellular molecules involved in TLR signaling are a new class of TLR inhibitors with promising preliminary data awaiting further clinical validation.

## 1. Toll-like Receptors: Structure, Signaling, Regulation

In 1997, a human homolog of the *Drosophila* Toll protein was discovered to induce the activation of nuclear factor κB (NF-κB) and the production of proinflammatory cytokines and co-stimulatory molecules [1]. To date, the family of human Toll-like Receptors (TLRs) comprises 10 members (TLR1-10) [2]. Structurally, TLRs contain a ligand-binding extracellular domain and a cytoplasmic Toll/interleukin 1 receptor (TIR) homology domain that orchestrates intracellular signaling cascades [3]. These receptors operate as sentinels of “stranger” or “danger” signals, recognizing structure-conserved molecules of microbes and endogenous ligands released from damaged cells. TLRs are strategically localized either on the cell surface (TLR1, TLR2, TLR4, TLR5, TLR6, and TLR10) surveilling the extracellular space or in the endosomal compartments (TLR3, TLR7, TLR8, and TLR9) monitoring the intracellular space [2,4]. Cell-surface TLRs mainly recognize ligands derived from microbial membrane components including lipids, lipoproteins, and proteins. Endosomal TLRs recognize viral, bacterial, and self-nucleic acid fragments of double-stranded RNA (dsRNA; TLR3), single-stranded RNA (ssRNA; TLR7, TLR8), and single-stranded DNA (ssDNA; TLR9) [4].

Due to structural similarities, TLRs share common signaling principles and signaling molecules [2]. Upon ligand binding, TLRs homo- or hetero-dimerize, and then TIR domains facilitate the assembly of signaling complexes (signalosomes). Apart from TLR3, a critical step of the signaling cascade in TLRs is the assembly of a signalosome termed the “Myddosome”, comprised of myeloid differentiation factor 88 (MyD88) and members of the interleukin 1 receptor-associated kinase (IRAK) family [3]. Downstream of signalosomes, there is activation of the NF-κB and mitogen-activated protein kinase (MAPK) pathways that induce the production of a constellation of inflammatory mediators (e.g., proinflammatory cytokines, chemokines, tissue-damaging enzymes). Endosomal TLRs and TLR4 also activate interferon regulatory factors (IRFs), a family of transcription factors that induce the production of type I interferons (IFNs) [4]. The functional consequences of TLR activation are tightly regulated at the levels of TLR protein expression, proximal signaling, and chromatin accessibility for TLR-induced transcription factors [4,5,6]. Dysregulated TLR activation has been described in many rheumatic diseases [7,8,9]. Here, we focus on recent developments in the therapeutic targeting of TLR signaling cascades in Systemic Lupus Erythematosus (SLE) and Rheumatoid Arthritis (RA).

## 2. Pathogenetic Role of Nucleic Acid Sensing by Endosomal TLRs in SLE

The hallmark of SLE is the break of tolerance to self-nucleic acids and the production of antinuclear antibodies (ANAs) with specificities against double-stranded DNA (anti-dsDNA) and ribonucleoproteins (RNPs; anti-RNPs) [8]. Enhanced TLR7 signaling, triggered by nucleic acid-containing immune complexes (ICs), has emerged as a central event in the pathogenesis of SLE. Human genetics and studies in animal models indicate at least three distinct mechanisms of enhanced TLR7 signaling that may coordinate during SLE pathogenesis: (1) continuous engagement of TLR7 due to endosomal abundance of stimulating ligands [4,8], (2) hypersensitive TLR7 due to gain-of-function mutations in the *TLR7* gene that lower the activation threshold of TLR7 [10], and (3) increased expression of TLR7 due to a higher number of functional copies of the *TLR7* gene [11] or single-nucleotide polymorphisms (SNPs) that render TLR7 transcripts resistant to degradation [12].

Under physiologic conditions, the endosomal availability of nucleic acids is tightly regulated to prevent “inappropriate” activation of endosomal TLRs [4]. Regulation involves nucleic acid handling at the levels of release, clearance, receptor-mediated uptake, and export from endosomes. Nucleic acid handling is dysregulated in SLE and, as a result, there is increased endosomal availability of nucleic acids. Dysfunctional neutrophils extruding nucleic acid-containing neutrophil extracellular traps (NETs) [13] and defective digestion of nucleic acids have been observed in SLE [4]. Three receptor systems have been involved in the process of uptake and delivery of nucleic acids to the endosomes, operating in a cell type-specific manner. In B cells, DNA- and RNA-containing antigens obtain access to the endosomes via binding to the B-cell receptor (BCR) [14,15]. In other TLR7-bearing cells (plasmacytoid dendritic cells (pDCs), myeloid DCs (mDCs), monocytes, and macrophages), nucleic acid uptake is mediated by Fcgamma receptors (FcγRs) [16,17,18] and the receptor for advanced glycation end-products (RAGE) [19,20,21]. RAGE binds nucleic acids directly [20,21] or through the high-mobility group box 1 (HMGB1) protein [19]. TLR7 ligands are actively exported from the endosomes by a family of nucleoside transporters including solute carrier family 29 member 3 (SLC29A3). *Slc29a3-*/- mice display endosomal accumulation of nucleosides that drive enhanced TLR7 activation [22]. In Asian patients with SLE, an SNP (rs780669) in the *SLC29A3* gene was recently identified as a risk variant for SLE [23]. The mRNA levels of SLC29A3a were found to be lower in the monocytes of these Asian patients compared to healthy controls.

The recent discovery of a lupus-causing gain-of-function TLR7 variant (Y264H) [10] represents the most compelling and human-relevant evidence that enhanced TLR7 signaling is involved in SLE pathogenesis. The mutated tyrosine residue of the Y264H variant lies in the ligand-binding site of TLR7. This single amino-acid substitution increases the binding affinity of TLR7, specifically for guanosine-containing ligands, and notably raises the TLR7 sensitivity to otherwise non-stimulating ssRNAs. The functional consequences of lowering the threshold of TLR7 activation were revealed when the Y264H variant was introduced into mice that otherwise were not prone to lupus. This new mouse strain, named *kika*, spontaneously developed a lupus-like phenotype with proliferative glomerulonephritis, widespread lymphadenitis, ANAs, thrombocytopenia, and decreased survival [10]. Increased levels of MyD88 in splenocytes of *kika* mice are consistent with enhanced TLR7 signaling. Crossing of *kika* mice with MyD88-knock-out mice completely rescued the lupus-like phenotype, further supporting the role of the TLR7–MyD88 signaling axis in SLE pathogenesis.

Prior studies have identified enhanced TLR7 signaling due to a higher number of functional *TLR7* gene copies producing more copies of TLR7 mRNA and protein. A 4-megabase DNA locus, duplicated from the X chromosome and translocated to the Y chromosome, was discovered in the males of the SB/Le mice strain. Notably, when this locus was transferred by crossing or inserted via genetic engineering in various lupus-prone mice, it induced the exacerbation of disease phenotype in males [24]. Thus, this locus was named Y-linked autoimmune accelerator (Yaa). Carriers of Yaa have an additional copy of the *TLR7* gene (*TLR7* duplication), express higher cellular levels of TLR7 mRNA and protein, and display enhanced TLR7 signaling. The impact of the *TLR7* gene copy number in the development of lupus was further investigated using genetically engineered mice that carry varying copy numbers of the *TLR7* gene (*TLR7* gene dose ranged from 0 to 32 copies) [25]. In lupus-prone mice strains, a reduction of the *TLR7* copy number from 2 to 1 abrogated the autoimmune accelerator effect. In mice strains not prone to lupus, overexpression of TLR7 (introduction of over four copies of the *TLR7* gene) was sufficient to induce a spontaneous lupus-like phenotype (glomerulonephritis, ANAs, increased serum levels of inflammatory cytokines, and increased lethality) [25].

The concept that TLR7 dosage is positively associated with SLE-provoking signaling could be a potential explanation for the strong female bias observed in SLE, the 14-fold higher incidence of SLE in 47 XXY males (Klinefelter syndrome) compared to 46 XY males, and the lower incidence of SLE in 45 XO females (Turner syndrome) [26]. TLR7 is encoded by a gene on the X chromosome and is primarily expressed in pDCs, mDCs, monocytes/macrophages, and B cells. In 46 XX females, each cell randomly inactivates one of its two X chromosomes to equalize gene dosage (monoallelic expression) with 46 XY males. However, up to 30% of X-linked human genes escape X-chromosome inactivation (XCI) so that both alleles can be expressed simultaneously (biallelic expression) [27]. Single-cell analysis has recently demonstrated that a large proportion of pDCs, B cells, and monocytes from 46 XX women and 47 XXY Klinefelter males are biallelic for the *TLR7* gene [11]. Functional experiments indicate that the escape of the *TLR7* gene from XCI endows the biallelic B cells with a higher cellular expression of TLR7 and increased responsiveness to TLR7 ligands. In addition to copy number variations, increased TLR7 expression was found to be the result of decreased TLR7 mRNA degradation. An SNP (rs3853839) in the 3′ untranslated region (UTR) of TLR7 mRNA is a risk variant for SLE in Asians and was found to decrease the binding of miR3148 to TLR7 mRNA [12]. As a result, there is reduced TLR7 mRNA degradation and increased expression of TLR7, combined with a higher IFN-signature score in the peripheral blood mononuclear cells (PBMCs) of SLE patients.

The lupus-promoting functional consequences of enhanced TLR7 signaling result from the direct activation of the TLR7-bearing cell types [8]. In pDCs and mDCs, TLR7 activation induces migration to the sites of inflammation, propagation of autoimmunity, and tissue damage due to the secretion of type I IFNs, inflammatory cytokines, and chemokines [16,17]. In autoreactive B cells, B-cell-intrinsic and -extrinsic TLR7 signaling provides the necessary co-stimulation for proliferation and differentiation to plasma cells and the production of autoantibodies against RNA-containing antigens [15]. In monocytes, TLR7 induces monocyte subset-specific signaling, endowing distinct SLE-related pathogenic functions. In the inflammatory Ly6C^hi^ monocyte subset, TLR7 drives the differentiation to inflammatory hemophagocytes with a high phagocytic capacity that may contribute to the inflammatory cytopenia and macrophage activation syndrome observed in SLE [28]. In the CD14^dim^ monocyte subset, nucleic acid sensing by TLR7 induces the production of CCL3 and TNF [29]. CD14^dim^ patrolling monocytes are present in the glomeruli of lupus patients. In lupus glomerulonephritis, deposited nucleic acid-containing ICs induce the TLR7-mediated activation of CD14^dim^ monocytes. Another study has demonstrated that TLR7 signaling protects pDCs and B cells from glucocorticoid-induced cell death [30], suggesting that TLR7 activation is not only disease-promoting but may also confer resistance to the standard-of-care treatment with glucocorticoids. In this context, pharmacologic inhibition of TLR7 signaling has emerged as an attractive treatment and steroid-sparing approach for SLE.

## 3. Therapeutic Targeting of Endosomal TLRs in SLE

**Figure 1:**
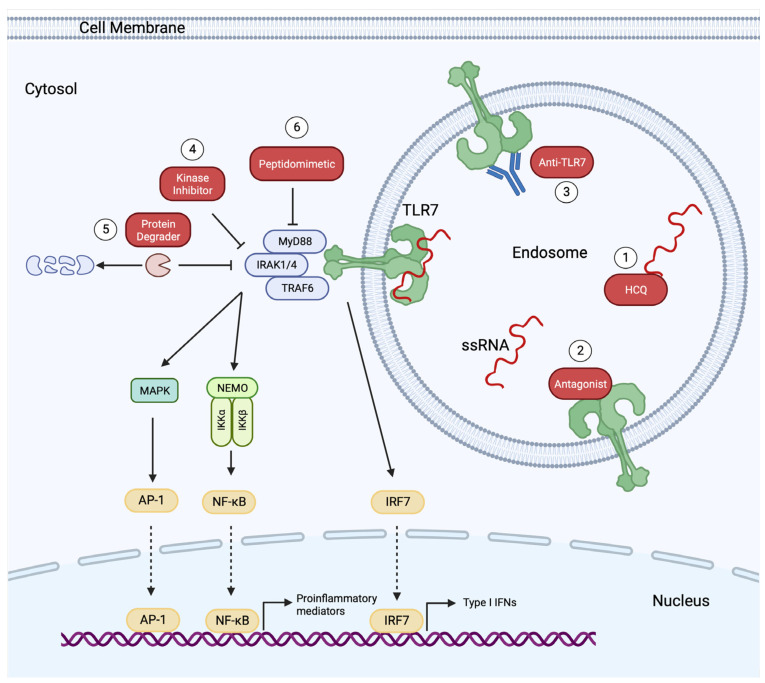
Pharmacologic inhibition of TLR7 pathway. TLR7 inhibitors either prevent the binding of ligands to TLR7 (1–3) or block downstream signaling cascades (4–6). Hydroxychloroquine (HCQ) prevents TLR7 engagement by sequestering TLR ligands through direct binding (1). TLR7 antagonists (ligand analogs) occupy TLR7 binding sites without inducing downstream signaling (2). Monoclonal antibodies against TLR7 (anti-TLR7) mask the ligand-binding sites (3). Small molecules inhibit the kinase activity of IRAKs (4). Protein degraders induce targeted ubiquitination and proteasome degradation of ΙRAΚ4 (5). Peptidomimetics inhibit the assembly of “Myddosome” (6). TLR, Toll-like Receptor; ssRNA, single-stranded RNA; MyD88, myeloid differentiation factor 88; IRAK1/4, interleukin 1 receptor-associated kinase 1/4; TRAF6, tumor necrosis factor receptor (TNFR)-associated factor 6; MAPK, mitogen-activated protein kinase; NEMO, nuclear factor κB essential modulator; IKKα, inhibitory κB kinase alpha; IKKβ, inhibitory κB kinase beta; NF-κB, nuclear factor κB; AP-1, activating protein-1; IRF7, interferon regulatory factor 7; IFNs, interferons. This figure was created using the tools provided by BioRender.com (accessed on 5 January 2024).

Several TLR7 inhibitors have been developed in the last decade. Based on their mechanism of action, TLR7 inhibitors are classified into the following categories (Figure 1): (1) molecules that sequester TLR ligands, preventing their binding to endosomal TLRs [31]; (2) oligonucleotide-based antagonists [32]; (3) small-molecule antagonists [33]; (4) monoclonal antibodies (mAbs) against TLR7 (anti-TLR7) [34]; and (5) small molecules inhibiting kinases [35] or targeting adaptor/scaffolding molecules downstream of TLRs [36]. Below, we describe TLR7 inhibitors that have passed the pre-clinical stage of in vitro and in vivo validation and we highlight those that have entered clinical development in humans (Table 1).

A proof of concept regarding the therapeutic potential of endosomal TLR inhibition in SLE comes from the effectiveness of antimalarial drugs (hydroxychloroquine (HCQ), chloroquine, and quinacrine) in SLE patients [37]. Inhibition of endosomal TLR signaling is one mechanism that explains, at least in part, the clinical benefit of antimalarials in SLE [38]. Due to high lipophilicity, antimalarial compounds can pass through the cell membranes and accumulate in lysosomes and endosomes (lysosomotropism). Within the endosomal compartments, antimalarial drugs directly bind and sequester nucleic acids, preventing ligation to endosomal TLRs and inhibiting downstream signaling [39]. This discovery has opened the avenue of steric inhibition of endosomal TLRs by synthetic chemical compounds that interact physically either with TLR ligands or with TLRs.

The discovery of short DNA immunoregulatory sequences (IRS) led to the development of oligonucleotide-based inhibitors of endosomal TLRs [40,41]. IRS 954 (DV-1079; dual TLR7/9 antagonist) and IRS 661 (TLR7 antagonist) have shown effectiveness in murine models of SLE [42]. Chemically modified oligonucleotides were developed subsequently, including immune-modulatory oligonucleotides (IMOs) such as IMO-8400 (Bazlitoran; triple TLR7/8/9 antagonist), IMO-9200 (triple TLR7/8/9 antagonist), and IMO-3100 (dual TLR7/9 antagonist) [33,43]. Despite the promising results of the above oligonucleotide-based antagonists in murine models of lupus, the clinical development of DV-1179 (a dual TLR7/9 antagonist) was halted after failing to achieve pharmacodynamic effectiveness in SLE patients [43] and none of these inhibitors is currently in clinical development for SLE (Table 1).

Recent studies have revealed the structural requirements for effective TLR7 ligation and have shown that TLR7 is a dual receptor that recognizes oligonucleotide-based ligands and small-molecule ligands with distinct binding sites [44,45,46]. These studies have paved the way for the rational design of small molecules, synthesized by chemical switches on the scaffold of TLR7 agonists (chemotypes) that retain TLR7-binding capacity but eliminate downstream signaling (antagonistic ligand mimetics) [33]. Depending on their fine structure, these chemotypes display variable selectivity (single- vs. double- vs. triple selectivity for TLR7, TLR8, and TLR9) and antagonistic potency. Four small molecules, dual inhibitors of TLR7 and TLR8 (Afimetoran/BMS-986256, Enpatoran/M5049, MHV370, and E6742), have successfully passed the stage of preclinical validation in various murine models of lupus and recently entered the early phases of clinical development in humans [47,48,49,50,51,52,53,54,55,56,57]. CPG-52364 (triple TLR7/8/9 antagonist) has been evaluated only in a phase I study (NCT00547014) with no further clinical development [33].

Recent evidence suggests that TLR7 shuttles not only between the endoplasmic reticulum and endosomal compartment but also to the cell surface [58]. TLR7 on the surface of the cell becomes accessible to inhibitory anti-TLR7 mAbs and forms TLR7/anti-TLR7 complexes that are internalized to endosomal compartments. The gradual accumulation of these complexes results in endosomes with TLR7 molecules covered by mAbs, incapable of recognizing nucleic acids [59]. The anti-TLR7 mAbs inhibit TLR7 responses in B cells, DCs, macrophages, and Ly6C^low^ patrolling monocytes and ameliorate serologic and pathologic manifestations of lupus in mice [60]. DS-7011a is an anti-TLR7 mAb that has shown ex vivo suppression of cytokine production by TLR7-stimulated PBMCs [61]. In a phase 1 single ascending dose study (NCT05203692), DS-7011a was well tolerated by healthy volunteers and it is now under evaluation in an ongoing phase 1b/2 study (NCT05638802) in patients with Systemic and Cutaneous Lupus Erythematosus [62,63].

Targeting downstream signaling molecules is another promising strategy for the therapeutic inhibition of endosomal TLRs. In the context of SLE, TLR7 ligation by self-nucleic acids results in the formation of the “Myddosome”, a signaling complex comprised of MyD88, IRAK1, and IRAK4 (Figure 1) [3]. Although numerous kinase inhibitors, with single specificity for IRAK4 (IRAK4i) or dual specificity for IRAK1 and IRAK4 (IRAK1/4i), are under in vitro testing and preclinical validation in animal models, only a few have entered clinical development in human phase I/II studies [35]. Among them, Edecesertib (GS-5718; IRAK4i) [64,65], Zimlovisertib (PF-06650833; IRAK4i) [66,67,68], and R835 (IRAK1/4i) [69,70,71] have shown promising effectiveness in murine models of lupus, ex vivo proof-of-mechanism in human PBMCs, and favorable safety in phase I studies. Edecesertib is currently in phase II (NCT05629208) for cutaneous lupus erythematosus. In addition to the kinase inhibitors, various peptidomimetic small molecules that inhibit TLR signaling by disrupting the assembly of “Myddosome” are under development [36].

## 4. Pathogenetic Role of TLR4 in RA

Germ-free conditions decrease the incidence and severity of inflammatory arthritis in susceptible animal models [72]. In humans, alterations in oral and intestinal microbiota (dysbiosis) have been involved in the pathogenesis of RA [73,74]. The link between oral dysbiosis (due to the predominance of periodontal pathogens such as *Porphyromonas gingivalis* and *Prevotella intermedia*) leading to periodontitis and RA has been supported by extensive evidence [73]. In addition, adjuvant administration is a typical methodology to provoke arthritis in animal models [75]. Activation of TLR pathways is a potential mechanism that links dysbiosis and adjuvants with the development of synovial inflammation. Animal models indicate a role of TLR4 in the propagation of inflammatory arthritis. Mice with a mutant defective TLR4 resolved faster serum transfer arthritis. In a model of collagen-induced arthritis, global knock-out of *TLR4*, although it had no inhibitory effect in the systemic production of proinflammatory mediators and anti-collagen antibody production, reduced the incidence and severity of arthritis, protected from cartilage damage, and decreased the levels of antibodies against citrullinated peptides (anti-CCP) [76]. These findings in animal models suggest that systemic events are TLR4-independent, while local joint inflammation and cartilage damage are, at least in part, TLR4-dependent.

Evidence from human studies provides additional support for the role of TLR4 in RA pathogenesis. SNPs in the *TLR4* gene have been associated with disease susceptibility, severity, progression, and prognosis [77,78,79]. Increased synovial expression of TLR4 [80,81,82], together with a dysregulated miRNA network that may influence TLR4 activity [83], has been described in patients with RA. The concept of TLR4 as a local amplifier of synovial inflammation and joint destruction is further supported by the observation of abundant endogenous TLR4 ligands within the inflamed joint [84,85]. The release of endogenous TLR4 ligands is the result of cell activation and local tissue damage. Continuous engagement of the overexpressed TLR4 by endogenous ligands in innate immune cells and fibroblast-like synoviocytes (FLSs) induces the production of inflammatory cytokines and tissue-destructive enzymes that fuel synovial inflammation and propagate joint destruction [86,87]. CD8+ cells expressing high levels of TLR4 with robust capacity of cytokine production in response to lipopolysaccharides (LPSs) were identified in the bloodstream of RA patients [88]. Notably, surface expression of TLR4 in CD8+ cells was directly correlated with disease activity. The unconventional expression of TLR4 in a subset of activated lymphocytes suggests that endogenous TLR4 ligands may contribute to RA pathogenesis by direct activation of TLR4-bearing cell subsets beyond innate immunity.

## 5. Therapeutic Targeting of TLRs in RA

**Figure 2:**
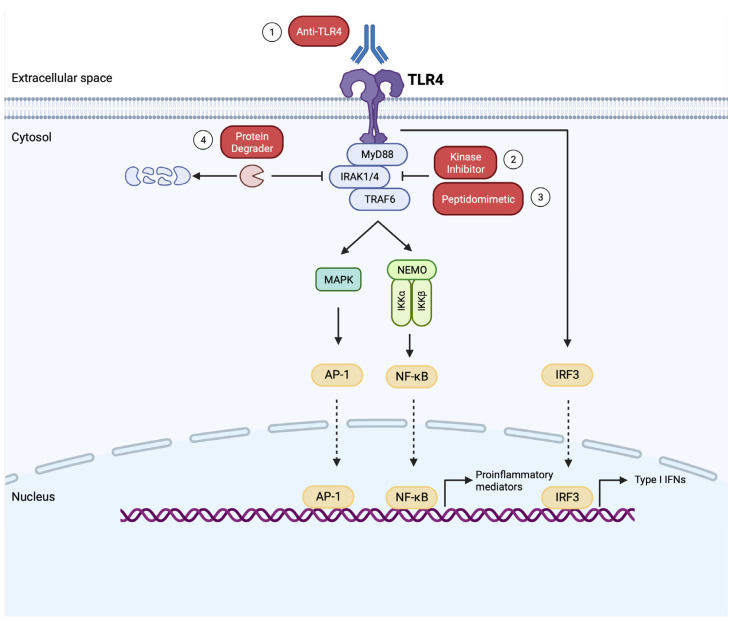
Pharmacologic inhibition of TLR4 pathway. (1) Monoclonal antibodies against TLR4 (anti-TLR4) mask the ligand-binding sites. (2) Small molecules inhibit the kinase activity of IRAKs. (3) Peptidomimetics and small molecules inhibit the assembly of “Myddosome”. (4) Protein degraders induce targeted ubiquitination and proteasome degradation of IRAK4. TLR, Toll-like Receptor; MyD88, myeloid differentiation factor 88; IRAK1/4, interleukin 1 receptor-associated kinase 1/4; TRAF6, tumor necrosis factor receptor (TNFR)-associated factor 6; MAPK, mitogen-activated protein kinase; NEMO, nuclear factor κB essential modulator; IKKα, inhibitory-κB kinase alpha; IKKβ, inhibitory-κB kinase beta; NF-κB, nuclear factor κB; AP-1, activating protein-1; IRF3, interferon regulatory factor 3; IFNs, interferons. This figure was created using the tools provided by BioRender.com (accessed on 5 January 2024).

Therapeutic strategies for inhibiting the TLR4 pathway in RA are summarized in Figure 2 and Table 1. Despite the evidence from preclinical mechanistic studies and human genetics about the potential involvement of TLR4 in the perpetuation of RA synovitis, NI-0101, a humanized mAb against TLR4, failed to improve arthritis in RA patients with an inadequate response to methotrexate [89]. Additional therapeutic modalities specifically targeting TLR4 signaling are under development and are expected to shed light on the actual role of the TLR4 pathway in human RA. For example, TAK-242 (Resatorvid) is a cell-permeable small molecule that selectively binds to Cys747 of the intracellular domain of TLR4, disrupting the interaction with adaptor proteins and inhibiting downstream signaling [90]. In a recent preclinical study, TAK-242 was proven effective in an animal model of inflammatory arthritis [91].

Emerging evidence implicates additional members of the TLR family in the pathogenesis of RA, suggesting a redundancy in TLR signaling. SNPs in TLR2, TLR3, TLR8, and TLR9 have been associated with RA [76]. Increased expression of TLR1, TLR2, TLR3, TLR7, and TLR8 has been described in RA synovium [80,81,82,87]. Serum amyloid A (SAA), an acute phase reactant produced during synovitis, was identified as an endogenous ligand for TLR2, mediating proinflammatory and angiogenic effects [92]. TLR2 was also found to promote FLS metabolic dysfunction [93], migration, and invasiveness [94]. An anti-TLR2 mAb (OPN301) inhibited ex vivo cytokine production by synovial tissue explants [95]. Notably, accumulating evidence reveals a potential role of TLR7 in RA. Expression levels of TLR7 in RA monocytes were strongly correlated with disease activity [96]. Endogenous TLR7 ligands (ssRNA and miR-let7b) were elevated in RA synovial fluid and the miR-Let7b/TLR7 pathway was found to foster metabolic malfunction in RA macrophages and FLSs, promote osteoclastogenesis, and potentiate murine synovitis [96,97]. Altogether, these observations indicate synergistic cooperation between TLRs overexpressed in the RA synovium that goes beyond TLR4. Pharmacologic targeting of molecules that integrate signaling downstream of multiple TLRs (e.g., IRAK inhibitors (IRAKi) and peptidomimetics targeting the “Myddosome”) are under development and will allow the broader inhibition of TLR pathways compared to TLR4 inhibition alone [98,99,100,101,102,103].

## 6. Future Perspectives

Evidence from animal models suggests that the TLR9 pathway has a controversial role in SLE, with studies indicating a protective impact [104]. In addition, distinct endosomal TLRs are required for different autoantibody specificities: TLR7 signaling promotes antibodies against RNA-containing antigens [15], whereas TLR9 signaling induces antibodies against DNA [14]. SLE displays large clinical and serologic heterogeneity [105], and it is worth investigating in future clinical trials whether TLR7-specific inhibition might be a more appropriate treatment choice, especially for patients with antibodies against RNA-containing antigens. This hypothetical concept that the serologic profile of SLE patients might be used as a biomarker to predict responsiveness to TLR7-specific inhibition opens an opportunity for a precision medicine approach in the management of SLE.

Novel strategies for therapeutic inhibition of TLRs are under development for SLE and RA. Protein degraders targeting the “Myddosome” have emerged recently as promising alternatives to kinase inhibitors and peptidomimetics (Table 1). KT-474 (SAR444656) is a proteolysis-targeting chimera (PROTAC) composed of an E3 ligase-binding portion linked to an IRAK4-binding portion [106]. This heterobifunctional small molecule links the E3-ligase cereblon (CRBN) to IRAK4, leading to the ubiquitination and proteasomal degradation of IRAK4. Preliminary data from a phase 1 study in patients with Hidradenitis Suppurativa (HS) and Atopic Dermatitis (AD) suggest a promising safety and pharmacodynamic profile. Notably, the IRAK4 kinase inhibitor Zimlovisertib has failed to show clinically significant effectiveness in HS and RA (Table 1). The theoretical advantage of IRAK4 degraders over the IRAK4 kinase inhibitors is that protein depletion abrogates both the kinase activity and the scaffolding function of IRAK4 [106]. Additional PROTACs targeting IRAK4 (GS-6791/NX-0479) or other signaling components of TLRs are under development. Further studies are required to investigate whether protein degraders will be proven safe and more efficacious than kinase inhibitors and peptidomimetics. Finally, targeting the mechanisms regulating neutrophil extracellular trap activation and release (NETosis) [107], the clearance of nucleic acids [4], the receptor-mediated shuttling of nucleic acids from the extracellular space to the endosomes [14,15,16,17,18,19,20,21], the chaperone molecules that control TLR trafficking [108], and the miRNA network that regulates TLR signaling [83] represents promising future approaches for the therapeutic manipulation of TLRs.

## Figures and Tables

**Table 1:** TLR inhibitors in clinical development for autoimmune/inflammatory diseases. TLR, Toll-like Receptor; IMO, immune modulatory oligonucleotide; SLE, Systemic Lupus Erythematosus; CLE, Cutaneous Lupus Erythematosus; RA, Rheumatoid Arthritis; DM, Dermatomyositis; SS, Sjogren’s Syndrome; MCTD, Mixed Connective Tissue Disease; IBD, Inflammatory Bowel Disease; AD, Atopic Dermatitis; HS, Hidradenitis Suppurativa; IRAK1/4, interleukin 1 receptor-associated kinase 1/4.

Class of TLR Inhibitors	Name	Target	Clinical Development
Monoclonal Antibodies	DS-7011a	TLR7	SLE, CLE: phase 2 (NCT05638802)
	NI-0101	TLR4	RA: failed (NCT03241108)
TLR Ligand Sequestration Molecules (Antimalarial Drugs)	Hydroxychloroquine (HCQ)	Endosomal TLRs	SLE, RA: in clinical use
Oligonucleotide-based Antagonists	DV-1179	TLR7/9	SLE: failed
	IMO-3100	TLR7/9	Psoriasis: completed phase 2 (NCT01622348) No updates since 2018
	IMO-8400	TLR7/8/9	Psoriasis: completed phase 2 (NCT01899729) DM: completed phase 2 (NCT02612857) No updates since 2019
	IMO-9200	TLR7/8/9	Discontinued
Small-molecule Antagonists	CPG-52364	TLR7/8/9	Discontinued after phase 1 (NCT00547014)
	Afimetoran (BMS-98652)	TLR7/8	SLE: phase 2b (NCT04895696)
	Enpatoran (M5049)	TLR7/8	SLE, CLE: phase 2 (NCT05162586)
	MHV370	TLR7/8	Healthy Adults: well tolerated in phase 1 (EudraCT number 2017-004559-21) SS & MCTD: Phase 2 discontinued by Sponsor (NCT04988087)
	E6742	TLR7/8	SLE: phase 1/2 (NCT05278663)
Kinase Inhibitors	Edecesertib (GS-5718)	IRAK4	RA: withdrawn (NCT05165771) IBD: discontinued CLE: phase 2 (NCT05629208)
	Zimlovisertib (PF-06650833)	IRAK4	RA: failed (NCT02996500)
	BAY1830839	IRAK4	Phase 1: NCT03965728, NCT03540615, NCT05003089
	Zabedosertib (BAY1834845)	IRAK4	AD: phase 2 Active, not recruiting (NCT05656911)
	EVO101	IRAK4	AD: discontinued (NCT05579899)
	R835	IRAK1, IRAK4	Phase 1
Protein Degraders	KT-474 (SAR444656)	IRAK4	AD: phase 2 (NCT06058156) HS: phase 2 (NCT06028230)
	GS-6791 (NX-0479)	IRAK4	Planned

## Data Availability

Data are contained within the article.
